# DFT Structural and UV–Vis Spectral Insights into Photosensitivity of Vandetanib: A Dual EGFR/SARS-CoV-2 Mpro Inhibitor

**DOI:** 10.3390/ph18091297

**Published:** 2025-08-29

**Authors:** Feng Wang, Vladislav Vasilyev

**Affiliations:** 1 School of Science, Computing and Emerging Technologies, Swinburne University of Technology, Melbourne, VIC 3122, Australia; 2 National Computational Infrastructure, Australian National University, Canberra, ACT 0200, Australia; vvv900@nci.org.au

**Keywords:** vandetanib, EGFR tyrosine kinase, SARS-CoV-2 mpro dual inhibitor, photosensitivity, density functional theory (DFT), charge-transfer excitation

## Abstract

**Background**: Vandetanib is a clinically approved epidermal growth factor receptor (EGFR) tyrosine kinase inhibitor (TKI) used in the treatment of medullary thyroid cancer. Recent studies have also suggested potential activity against the SARS-CoV-2 main protease (Mpro), indicating dual therapeutic relevance. However, its clinical use is limited by photosensitivity side effects, the molecular basis of which remains poorly understood. This study aims to elucidate the conformational, spectroscopic, and electronic properties of vandetanib underlying its photoreactivity. **Methods**: Density functional theory (DFT) was employed to explore vandetanib’s conformational landscape, electronic structure, and spectroscopic behavior. Low-energy conformers were identified and compared with experimental crystal and NMR data. Time-dependent DFT (TD-DFT) calculations were used to simulate UV–Vis absorption spectra and assign key electronic transitions. **Results**: Eight low-energy conformer clusters, including the global minimum structure, were identified. The global minimum was validated by consistency with crystal and experimental NMR data, emphasizing the role of conformational averaging. TD-DFT simulations successfully reproduced the two main UV–Vis absorption bands, with the primary band (~339 nm) assigned to a HOMO–1 → LUMO charge-transfer excitation between the N-methyl piperidine and quinazoline rings, pinpointing a structural contributor to photoreactivity. Additionally, the N-methyl piperidine ring was identified as a major metabolic hotspot, undergoing multiple biotransformations potentially linked to phototoxicity. **Conclusions**: This study provides molecular-level insights into the structural and photophysical origins of vandetanib’s photosensitivity. The findings improve understanding of its adverse effects and can inform the safer design of EGFR-targeting drugs with reduced phototoxic risks.

## 1. Introduction

The rapid emergence and evolution of SARS-CoV-2 variants during the COVID-19 pandemic have exposed a critical vulnerability in current antiviral strategies. Viral mutations can significantly diminish the efficacy of vaccines and monoclonal antibodies, highlighting the urgent need for quickly deployable antiviral agents [1]. There is a particular demand for orally available therapeutics that are suitable for outpatient use and capable of targeting both viral replication and the dysregulated immune response, including the cytokine storm that often leads to severe complications such as acute respiratory distress syndrome (ARDS) and multi-organ failure. Although antivirals such as Remdesivir [2] and Paxlovid [3] have been approved or authorized for emergency use, treatment options remain limited, especially during the immunopathogenic phase of the disease. In this context, drug repurposing—the strategic application of existing U.S. Food and Drug Administration (FDA)-approved drugs for new indications—has emerged as a time-efficient and cost-effective approach to antiviral discovery [1]. Repurposed drugs offer known safety profiles, established pharmacokinetics, and the possibility of expedited clinical translation, making them attractive candidates in the race to contain fast-mutating viruses like SARS-CoV-2 [1].

Vandetanib (VTB, ZD6474) has gained attention as a candidate for repurposing due to its dual therapeutic potential. Originally developed by AstraZeneca and approved by the U.S. FDA in 2011 for the treatment of locally advanced or metastatic medullary thyroid carcinoma [4,5], VTB is a multi-targeted tyrosine kinase inhibitor (TKI) [6]. It acts on several key targets—epidermal growth factor receptor (EGFR), vascular endothelial growth factor receptor (VEGFR), and rearranged during transfection (RET) tyrosine kinase—and is used clinically in oncology, including non-small-cell lung carcinoma [6,7].

**Figure 1:**
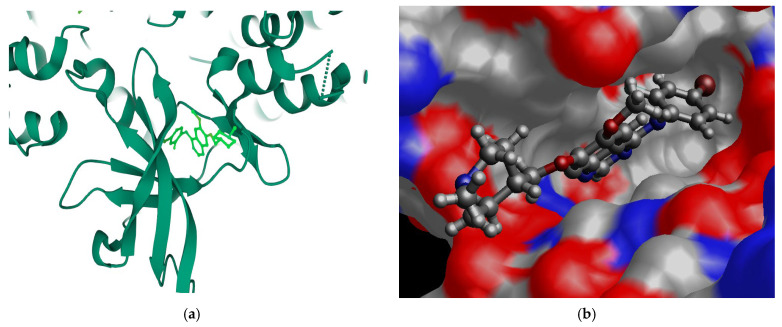
(**a**) The complex of 2ivu co-crystallized with vandetanib (C_22_H_24_BrFN_4_O_2_, N-(4-Bromo-2-fluorophenyl)-6-methoxy-7-((1-methylpiperidin-4-yl)methoxy)quinazolin-4-amine) (PDB: https://www.rcsb.org/). VTB occupied the ATP binding pocket of EGFRL858R/T790M/C797S [12]. (**b**) The local area complex area of 2ivu with VTB is illustrated. The color scheme is the atom types, i.e., red—oxygens, blue—nitrogens, and grey hydrogens & carbons.

In cancer models, VTB demonstrates strong kinase inhibition, with IC_50_ values of 19.76 nM for EGFR and 33.26 nM for VEGFR [8]. It also shows cytotoxicity in EGFR-overexpressing A431 and H1975 lung cancer cells (IC_50_ = 0.85 μM and 4.81 μM, respectively) [9]. Structurally, VTB features a quinazoline-based scaffold—a hallmark of 4-anilinoquinazoline class inhibitors—contributing to its broad-spectral activity [10]. Figure 1a shows the structure of the complex co-crystallized with VTB (2ivu) [11], which occupies the ATP binding pocket of EGFRL858R/T790M/C797S. The N-methyl piperidine and 4-anilinoquinazoline core of VTB form a hydrogen bond with Met793 in the hinge region [12] Figure 1b presents the local pocket of the complex of 2ivu with the VTB.

Beyond oncology, VTB has shown significant antiviral and immunomodulatory activity against SARS-CoV-2. In vitro studies demonstrated that VTB inhibits viral replication in A549-hACE2 and Caco-2 cells with an IC_50_ of 0.79 μM and no apparent cytotoxicity [13]. In vivo, treatment with VTB reduced levels of key inflammatory cytokines (IL-6, IL-10, and TNF-α) and chemokines (CCL2, CCL3, and CCL4) and alleviated lung inflammation in infected transgenic mice, although viral titers were not significantly lowered [13]. These findings suggest that VTB may exert its antiviral benefit through host-directed immunomodulation, a mechanism complementary to direct-acting antivirals. Moreover, VTB has also demonstrated broad-spectrum activity against SARS-CoV-2 variants. Shin et al. [14] reported that VTB exhibited the highest antiviral potency among tested FDA-approved EGFR inhibitors, including gefitinib, olmutinib, erlotinib, lapatinib, bosutinib, cabozantinib, icotinib, vendetinib, and brigatinib, against multiple variants of concern, including Alpha, Beta, Delta, and Omicron, reinforcing its promise in COVID-19 therapeutics. In addition to its pharmacological versatility, VTB undergoes CYP3A4-mediated metabolic transformations, forming N-desmethylvandetanib, a metabolite with similar potency (IC_50_ ≈ 0.12 μM for EGFR) [15], and VTB-N-oxide, which is significantly less active (>50-fold) [15]. These metabolic pathways can be influenced by drug–drug interactions and patient-specific enzyme expression, emphasizing the need for a better understanding of VTB’s metabolic stability and bioavailability [15].

**Figure 2:**
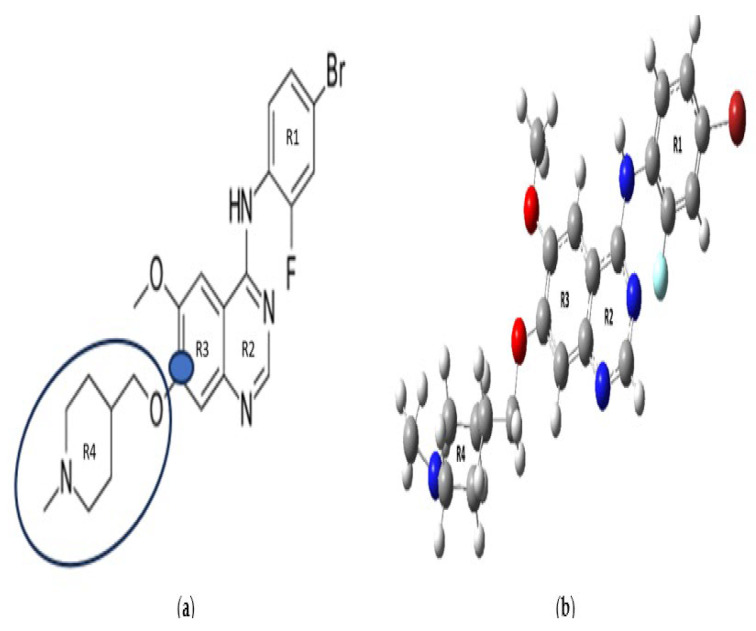
(**a**) Chemical structure of vandetanib. The potent C7-position (based on the IUPAC nomenclature) according to Hennequin et al. [31] is marked (blue circle) on the structure with an N-methyl piperidine ring. (**b**) The optimized ground electronic state structure of VTB using B3LYP/6-311++G(d,p) in DMSO using the PCM solvent model. Four rings—R_1_ for the bromofluorophenyl, R_2_ and R_3_ for the quinolinyl, and R_4_ for the N-methyl piperidinyl rings—are marked on the structure. Note that the numbers in (**b**) are the Gaussian labeling of the atoms. (The enlarged structure is presented, in Figure S1 in the Supplementary Materials (SM)).

The N-methyl piperidine ring, highlighted in the chemical structure of VTB (Figure 2a), is a cyclic tertiary amine, a functional group commonly found in drug molecules. Cyclic tertiary amines are known to undergo metabolic bioactivation, forming iminium intermediates, which are electrophilic and can react with nucleophiles such as cyanide (KCN) to form stable adducts [16]. These reactive intermediates are often implicated in idiosyncratic toxicities, including phototoxicity and QT interval prolongation, both of which are clinical concerns for VTB. Accordingly, incubation of VTB with rat liver microsomes (RLMs) in the presence of 1.0 mM KCN was performed to detect the formation of reactive metabolites. In both in vitro and in vivo phase I metabolism, the N-methyl piperidine ring undergoes several transformations, including N-oxide formation, N-demethylation, α-carbonyl formation, and α-hydroxylation, all of which can influence the drug’s stability and reactivity. Moreover, a phase II metabolic pathway involves direct glucuronidation of the N-methyl piperidine, further supporting its role as a metabolic hotspot [16]. These metabolic modifications likely contribute to the toxicity and reduced metabolic stability of VTB.

Beyond its metabolic vulnerability, the flexibility of the N-methyl piperidine ring plays an important role in ligand adaptability, facilitating VTB’s ability to adopt different conformations for binding distinct biological targets. In EGFR, the piperidine side chain may orient to optimize hydrophobic interactions within the kinase binding pocket. In contrast, for SARS-CoV-2 proteins such as the main protease (Mpro) or spike protein, the flexible piperidine moiety may adopt alternative conformations to fit different binding environments. Thus, this structural flexibility, while contributing to metabolic liability, may also underlie VTB’s dual-target inhibitory activity, enabling it to engage both human kinases and viral proteins through distinct conformational states [16].

Nuclear magnetic resonance (NMR) spectroscopy is one of the most widely used techniques for structural characterization of small-molecule TKIs such as VTB [17,18]. By providing detailed insights into chemical environments, molecular conformations, and dynamic interactions, NMR facilitates the elucidation of structural features essential for quantitative structure–property relationship (QSPR) studies. When combined with computational approaches such as density functional theory (DFT), these experimentally validated insights enhance the accuracy of QSPR models, aiding in the prediction of pharmacological and physicochemical properties of VTB and related compounds [19]. The N-methyl piperidinyl ring of VTB may contribute significantly to the unique functionality of the inhibitor [16], potentially influencing the high-energy π → π* transitions observed in many conventional anilino-quinazoline-based inhibitors such as PB153035 [20]. Several semi-empirical methods have been developed to investigate the structure of drug candidates. For instance, predictive tools exist for evaluating the structures of flavone derivatives that are either not naturally occurring or inaccessible by standard synthetic routes [21,22,23,24]. More recently, a machine learning (ML)-based NMR approach known as computer-assisted structure elucidation (CASE) has been introduced, targeting organic compounds containing no more than 10 non-hydrogen atoms [25]. These semi-empirical methods are generally fast and useful for the rapid estimation and characterization of potential drug structures. However, molecular structures are governed by quantum mechanics, which can place them beyond the predictive limits of such simplified models [26], especially for larger molecules like VTB (C_22_H_24_BrFN_4_O_2_), which contains 30 non-hydrogen atoms, well beyond the CASE method’s size limit.

Another important consideration is VTB’s photoreactivity. Studies have shown that UV irradiation can induce phototoxic effects in human keratinocytes, leading to oxidative stress and skin damage [27]. Its anilino-quinazoline core, which includes halogenated fluorophores, absorbs light in both the UVA (315–400 nm) and UVB (280–315 nm) regions, contributing to its photosensitive behavior [28,29]. Raising awareness of the photosensitizing potential of certain anticancer agents—including quinazoline-based inhibitors—and their clinical manifestations is critical for enabling the early identification of additional photosensitizing compounds and for preventing adverse phototoxic reactions [30].

Given these multifaceted therapeutic and chemical properties, it is crucial to investigate VTB at both the molecular and electronic levels. In this study, we employ density functional theory (DFT) and time-dependent DFT (TD-DFT) to simulate and analyze the NMR and UV–vis spectra of VTB in relevant solvents. These computational approaches allow us to examine the electronic structure, solvent interactions, and spectroscopic behavior of VTB, linking them to its physicochemical and pharmacological properties. By integrating quantum chemical analysis with pharmacological data, this work provides mechanistic insights into VTB’s dual therapeutic potential in cancer and antiviral treatments. As a result, accurate, quantum mechanics-based, and benchmarked DFT calculations are essential for a comprehensive understanding of VTB’s behavior and activity.

## 2. Results and Discussion

### 2.1. Low-Energy Conformers of Vandetanib in Solvent

**Figure 3:**
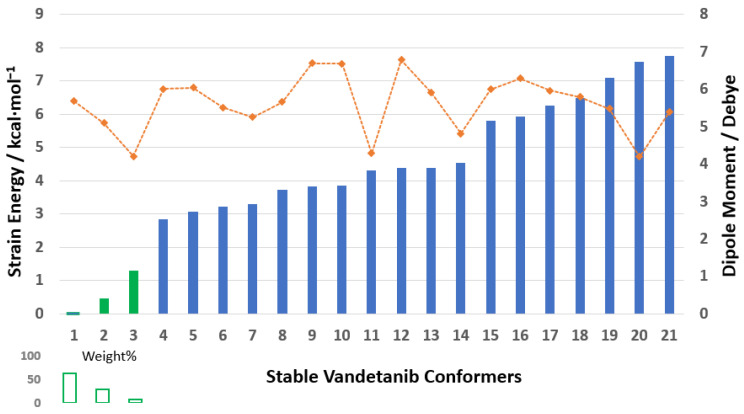
The strain energy (kcal·mol^−1^) of the partially optimized VTB conformers with a cut off at 7.758 kcal·mol^−1^ (blue columns) and their corresponding dipole moments (in Debye, orange), based on B3LYP/6-311++G(d,p) level of theory. The detailed results are in Table S1 of the SM.

All geometries of VTB using cluster sampling [32] were optimized using the DFT- based B3LYP/6-311++G(d,p) level of theory in DMSO solvent. The PrefQMCon program clustered the generated stable structures into 21 conformer groups, of which only the first eight VTB clusters exhibit populations exceeding 0.1% at room temperature (293.0 K) in DMSO. The energy differences between the global minimum structure and the energies of stable conformers of local minimum structures are due to conformational strain energy [32]. The strain energy of VTB conformers with a cut off at 7.758 kcal∙mol^−1^ is given in Figure 3. (The details are given in Table S1 in the SM). Notably, when the strain energy exceeds 7 eV, very few conformers are populated at 293.0 K according to the canonical partition function.

The three lowest-energy conformers of VTB, shown in green in Figure 3, account for approximately 98.21% of the conformational population at room temperature (293.0 K). The remainder of this study focuses on these dominant conformers, particularly the global minimum (Van1), which alone contributes nearly 63% to the overall Boltzmann distribution. Dipole moments vary significantly among the conformers, ranging from 4.0 to 8.0 Debye, which highlights the sensitivity of this anisotropic property to molecular conformation. Notably, among the dominant conformers, dipole moments tend to decrease as the conformational strain energy increases, reflecting the relationship between structural compactness and electronic distribution.

**Figure 4:**
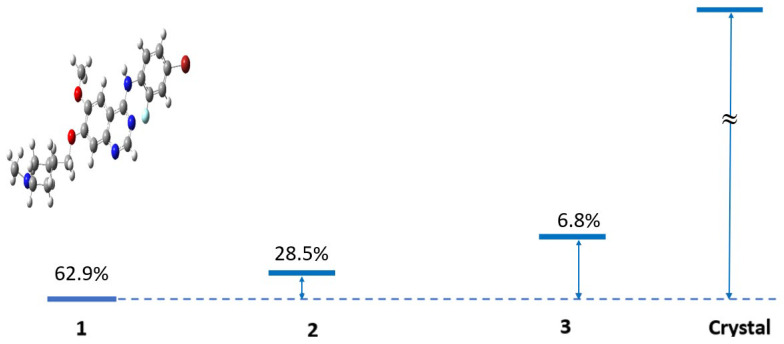
The three lowest-lying conformers of VTB, Van1 (the structure in the figure), Van2 (0.461 kcal∙mol^−1^ above Van1), Van 3 (1.293 kcal∙mol^−1^ above Van1), and the crystal structure [11] (370.23 kcal∙mol^−1^ above Van1). The strain energy and the weight (%) based on the canonical partition function at 293.0 K are also given at the B3LYP/6-311++G(d,p) level of theory. For more details, see Table S1 in the SM.

Figure 4 provides a structural comparison between the three lowest-energy conformers and the experimental crystal structure of VTB [11,14,33]. The quinazoline core of VTB is highlighted in orange color in the figure. A critical interaction site, carbon C7 on the 4-anilinoquinazoline fragment, is marked in blue in the inset, which was identified by Hennequin et al. [31] as a key position for EGFR inhibitor potency. In the most populated conformers (Van1, Van 2, and Van3), the fluorine atom is oriented on the opposite side of the C7 site connecting the N-methyl piperidine ring [31]. Although the bromo-fluorophenyl ring (R_1_) is not coplanar with the quinazoline core (R2 and R3), the fluorine atom consistently aligns with the dihedral angle between the bromo-fluorophenyl ring, and the quinazoline core is small (1.12°) in these dominant VTB conformers. In the crystal structure, this dihedral angle is 25.7°, suggesting a conformational rearrangement upon crystallization or binding.

These low-energy conformers of VTB presented in Figure 4 were reoptimized using the mPW1PW91/6-311++G(d,p) level of theory in DMSO to ensure accurate NMR predictions [34]. Key structural parameters of these conformers are summarized in Table 1. Compared to the crystal structure [11,14,33], all fully optimized geometries exhibit slightly shorter bond lengths and ring perimeters. This observation may stem from the absence of crystal packing effects in solvated DFT optimizations. A consistent trend is noted among the low-energy conformers of VTB regarding ring perimeter sizes: R_2_ (quinazoline moiety) < R_1_ (bromofluorophenyl) < R_3_ (quinazoline moiety) < R_4_ (methylpiperidinyl). This trend is chemically reasonable: R_2_ is a pyrimidine-type aromatic ring with multiple C–N bonds, contributing to a more compact geometry. In contrast, R_4_, which is a saturated piperidine ring with all single bonds, results in a more flexible and extended conformation.

The perimeters of R_1_ to R_3_ of VTB are in excellent agreement with those of structurally related EGFR TKIs, such as AG-1478 (8.37 Å, 8.21 Å, and 8.44 Å) [35] and Dacomitinib (8.30 Å, 8.16 Å, and 8.38 Å) [36]. Interestingly, the presence of dihalogen substitutions on the phenyl ring appears to induce a slight contraction of ring perimeters, with VTB (8.34 Å) and Dacomitinib (8.30 Å) both showing slightly shorter values than AG-1478 (8.37 Å) [35].

While isotropic geometric properties, such as ring perimeters, are nearly identical across the three low-energy conformers, the anisotropic properties like dipole moments and electronic spatial extension (shape) differ more significantly. The calculated dipole moments for Van1, Van2, and Van3 are 5.58, 4.99, and 5.97 Debye, respectively, corresponding to their electric spatial extents ⟨R^2^⟩ of 108,955.71, 99,622.38, and 49,763.67 atomic units. These variations reflect differences in molecular polarity, charge distribution, and shape, which could influence solvent interactions and NMR chemical shift predictions.

### 2.2. NMR Chemical Shifts of Low-Lying Conformers of Vandetanib

Beyond its routine analytical applications, NMR spectroscopy provides atom-specific information about molecular environments in solution. When combined with computational NMR simulations, it offers valuable insights into the preferred conformations and geometries of molecules under experimental conditions [26,37]. To validate the accuracy of quantum chemical methods for predicting NMR observables, we re-optimized the structure of VTB in DMSO. Based on these solvent-specific geometries, ^1^H-, ^13^C-, and ^15^N-NMR chemical shifts were calculated for the global minimum structure (Van1).

Among various NMR observables, ^1^H-NMR chemical shifts are particularly valuable for validating computational methods such as DFT, owing to their high experimental precision, reproducibility, and sensitivity to local electronic environments. Protons yield sharp, well-resolved signals, and the standardized ^1^H-NMR scale allows for direct comparison with theoretical predictions. The relatively narrow chemical shift range (0–12 ppm) enhances the detection of discrepancies between calculated and experimental values. In addition, ^1^H-NMR shifts are highly responsive to subtle electronic effects, including hydrogen bonding, where downfield shifts (higher ppm values) typically indicate deshielding of protons involved in intra- or intermolecular interactions [26,38].

For complex organic molecules such as VTB (C_22_H_24_BrFN_4_O_2_), ^1^H-NMR is generally measured with higher precision and reliability than ^13^C-NMR due to the higher sensitivity and natural abundance of ^1^H nuclei as well as more robust calibration protocols [17,39,40,41]. Moreover, DFT calculations capture local electronic effects on proton shifts more accurately than for carbon atoms, whose chemical shifts are often influenced by broader electronic delocalization and conformational dynamics, increasing both experimental uncertainty and computational complexity. In this study, experimental ^1^H-NMR data were first used to validate the computed chemical shifts and DFT functional. This validated functional was then applied to ^13^C-NMR shift calculations, allowing comparison with experimental data and providing insights into the inherent limitations and potential discrepancies of ^13^C-NMR measurements in thiscompound.

Experimentally determined NMR parameters are valuable for elucidating both the two-dimensional (2D) connectivity and three-dimensional (3D) structures of molecules [34]. Table S3 in SM presents the DFT-calculated ^1^H-NMR chemical shifts for three low-energy conformers of VTB (Van1, Van2, and Van3), alongside several experimental datasets reported by Al-Ghusn et al. [17], Smith et al. [37], Fei [40], and Brocklesby et al. [41] in methanol solvent. Although Fei’s study [40], conducted in 2016, is less recent than other measurements listed in Table S3, it employed a more advanced spectrometer. Specifically, Fei used a Bruker ARX-500 operating at 500.30 MHz, along with heteronuclear single quantum coherence (HSQC), ^1^H-^1^H correlation spectroscopy (COSY), and other 2D NMR experiments [40]. These 2D NMR spectra enabled Fei [40] to achieve confident and reliable assignments of the ^1^H-NMR chemical shifts. Therefore, Fei’s results [40] are used as a key reference for comparison in this study.

The calculated root mean square deviation (RMSD) between the theoretical and experimental ^1^H chemical shifts suggests that either the global minimum conformer Van1 or the Boltzmann-averaged low-energy conformers, denoted Van_w_, most likely represent the structure observed in Fei’s measurements [40], as they show the smallest RMSD of 0.29 ppm. Notably, the relative energies of Van1, Van2, and Van3 correspond well with the likelihood of their experimental detection. For comparison, the RMSDs between the calculated shifts and other available measurements are 0.21 ppm for Al-Ghusn et al. [17], 0.71 ppm for Smith et al. [37], and 0.32 ppm for Brocklesby et al. [41]. It is noted that although the proton NMR chemical shifts of measurements of Fei [40] are more accurate, it might have missed one aromatic proton NMR chemical shift signal between 7.1778 and 7.4643 ppm. Additionally, the second-to-last signal at 7.7893 ppm [40] is relatively lower than those reported by Smith et al. [37] and Brocklesby et al. [41] as well as the patent measurements of Tung [19].

**Figure 5:**
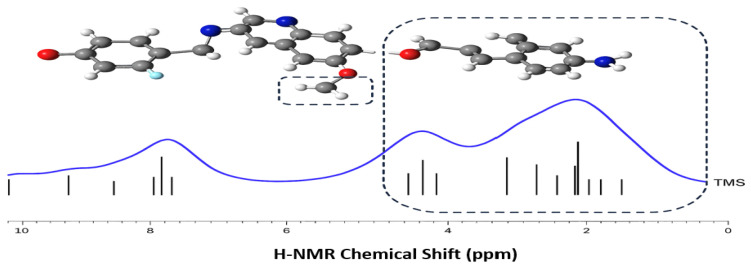
The ^1^H-NMR chemical shifts of VTB in DMSO. The chemical shifts for the aromatic protons (7 Hs) and the aliphatic protons (17 Hs) of VTB (Van1) are displayed in the dashed-lined box. All calculations were performed using mPW1PW91/6-311++G(d,p) in DMSO.

Figure 5 presents the ^1^H-NMR chemical shifts of the 24 protons in VTB. The spectrum is divided into two distinct regions: the aromatic protons (7 in total) appear downfield at δ_H_ > 7 ppm, while the remaining 17 aliphatic protons appear upfield at δ_H_ < 5 ppm. This clear separation arises from the distinct chemical environments of the protons and is consistent with the experimental spectrum reported by Fei [40]. Structurally, VTB can be divided into two main fragments: a relatively rigid aromatic region (the 4-anilinoquinazoline fragment, R1, R2 and R3) and a more flexible aliphatic region (the piperidine ring, R4), as illustrated in Figure 5. The aromatic protons are deshielded due to the electron-withdrawing nature of the conjugated aromatic system, resulting in downfield shifts. In contrast, the aliphatic protons are shielded by their saturated environments, leading to upfield shifts [19]. This clear distinction between the aromatic and aliphatic regions is well reflected in the NMR spectrum shown in Figure 5.

The consistently low RMSD values between the calculated and experimental ^1^H NMR chemical shifts confirm that the DFT approach employed in this study is sufficiently accurate for modeling the local electronic environments in VTB. Building on this, the same computational framework was extended to calculate the ^13^C NMR chemical shifts, where the choice of exchange functional was systematically evaluated. Table 2 summarizes the comparison of calculated carbon chemical shifts for the global minimum structure of VTB, using two exchange functionals, namely B3 and mPW91, combined with the same correlation functional, PW91, against three sets of experimental data. The RMSD values consistently indicate that the mPW91 exchange functional provides better agreement with experiment across all datasets. Specifically, for the experimental data of Al-Ghusn et al. [17], both functionals show comparable accuracy, with RMSD values of 3.20 ppm (B3PW91) and 3.15 ppm (mPW91/PW91). However, larger differences are observed with the datasets of Brocklesby et al. [41] and Fei [40]: B3PW91 gives RMSDs of 7.78 ppm and 5.70 ppm, respectively, whereas mPW91/PW91 achieves significantly lower values of 5.01 ppm and 3.57 ppm.

These results suggest that mPW91PW91 captures the electronic structure of VTB more accurately, particularly for the complex carbon environments found in the heterocyclic and aliphatic regions of the molecule. The improved performance of mPW91 may reflect its modified exchange treatment, which enhances the description of electron delocalization and polarization effects present in VTB. Use of mPW91PW91 may be a more reliable functional combination for predicting ^13^C chemical shifts for VTB, providing a better basis for QSPR studies and structural characterization of related tyrosine kinase inhibitors.

The ^13^C-NMR chemical shifts of VTB also reveal two distinct regions that reflect its structural features: aromatic carbons appear at δ_C_ > 120 ppm, while aliphatic carbons appear at δ_C_ < 100 ppm. The aliphatic region includes the –OCH_3_, –OCH_2_–, and –NCH_3_ groups and the piperidine ring (R_4_). This distribution of carbon chemical shifts in VTB is consistently observed across experimental studies. Table 2 compares the DFT-calculated δ_C_ values with experimental datasets from Al-Ghusn et al. [17], Fei [40], and Brocklesby et al. [41]. The calculations use two DFT exchange functionals (V_x_), B3 and mPW91, respectively, in combination with the correlation functional of PW91. Notably, Fei et al. employed the advanced DEPTQ (distortionless enhancement by polarization transfer with quadrature) technique, which provides enhanced spectral resolution and allows differentiation of CH, CH_2_, CH_3_, and quaternary carbons.

In DMSO, the combined DEPTQ and DEPT135 spectra of Fei [40] demonstrate improved sensitivity and resolution with more accuracy (small RMSD of 3.57 ppm). In contrast, Brocklesby’s measurements [41] appear to have been conducted under less controlled conditions, without reporting experimental details regarding dynamic averaging or rotameric effects from flexible bonds. This may have led to broader or less distinct peaks, reducing measurement accuracy. Quantitatively, this discrepancy is reflected in RMSD values between experimental and DFT-predicted ^13^C chemical shifts: 10.79 ppm (B3PW91) and 11.99 ppm (mPW1PW91) for Brocklesby [41] and 7.78 ppm (B3PW91) and 5.01 ppm (mPW1PW91) after thermal correction versus a significantly lower 5.70 ppm and 3.57 ppm for Fei [40], underscoring the latter’s superior agreement with theoretical data. Table 2 organizes the C-NMR chemical shifts as ordered from small to large values, as no assignment in the experiments is available except for a couple of distinct chemical shifts. Figure S4 in SM visualizes such differences based on each carbon site of VTB.

A closer examination of the calculated δ_C_ for individual carbon atoms highlights distinct performance patterns between the two exchange-correlation functional combinations across different experimental datasets, as shown in Figure S4 in the SM. For the Al-Ghusn et al. [17] dataset (blue), the B3PW91 functional shows larger deviations in the low δ_C_ region (saturated carbons below 100 ppm), whereas its predictions for carbons with δ_C_ > 100 ppm are generally more accurate, except for C21, C22, and C23, where significant discrepancies persist. In contrast, the mPW1PW91 functional provides improved accuracy across most carbons in this dataset, though notable deviations (greater than 5 ppm) remain for C13, C16, and C18.

For the Brocklesby et al. [41] data (green), B3PW91 achieves reasonable agreement for most carbons, with deviations generally within ±5 ppm. However, approximately one-third of the carbons show larger discrepancies, particularly C4, C8, C9, C12, C13, C14, C16, and C23. The mPW1PW91 functional performs better overall in this case, though significant deviations remain for C4, C8, C9, C14, C17, C18, C21, C22, and C23, indicating persistent challenges in accurately predicting shifts for certain carbon environments. In the Fei [40] dataset (orange), both functionals exhibit a similar overall level of deviation but with a notable observation: the sign of the deviation for many carbons is opposite between the two functionals, suggesting that the exchange functional influences the direction of the chemical shift error rather than its magnitude alone. This reflects subtle differences in how each functional treats electronic shielding and deshielding effects. The comparisons indicate that mPW1PW91 consistently provides better agreement across most carbon atoms. However, both functionals face challenges in accurately modeling shifts for certain carbons, particularly those in flexible aliphatic regions or at sites influenced by long-range electronic effects.

**Figure 6:**
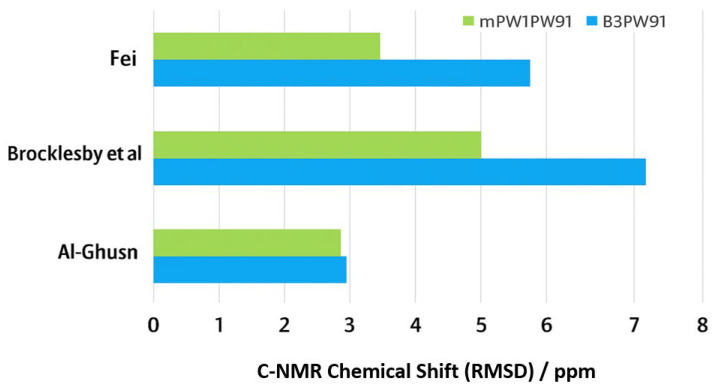
The RMSDs of the ^13^C-NMR chemical shifts based on DFT B3PW91/6-311++G(d,p) (blue) and mPW1PW91/6-311++G(d,p) (green) level of theory and the experiments of Al-Ghusn et al. [17], the corrected Brocklesby et al. [41], and Fei [40] (ppm). Note the RMSD results are the bottom lines in Table 2.

Due to the room-temperature conditions under which the measurements were taken, the rotational flexibility of single bonds likely led to thermally averaged ^13^C-NMR chemical shifts [42]. Specifically, in the piperidine ring, dynamic averaging appears to result in three observed carbon signals instead of five distinct chemical shifts in the experimental spectra, as shown in Table 2. These thermally averaged carbon pairs are indicated by blue and green dashed circles on the molecular structure in Figure S4 in the SM. When accounting for these averaged pairs, the corrected carbon chemical shifts for VTB—presented in Table 2—show a notable improvement in agreement with calculated values. However, the accuracy still falls short compared to the more advanced DEPT135 and DEPTQ techniques used by Fei [40], which provide superior spectral resolution and carbon type discrimination. Both DFT functionals, B3PW91 and mPW1PW91, show consistent accuracy compared with the three available experimental measurements. However, mPW1PW91 yields slightly more accurate results overall, as illustrated in Figure 6.

The discrepancies between calculated and experimental ^13^C- and ^1^H-NMR chemical shifts for VTB and related drug analogues can be attributed to several factors, including experimental temperature, sample purity, and the possible presence of multiple conformers under measurement conditions. In particular, room-temperature measurements allow for free rotation of flexible groups—such as terminal methyl (-CH_3_) groups—which leads to three-fold signal degeneracy due to rapid conformational averaging [42]. These dynamic effects can obscure or merge chemical shifts in the spectrum, complicating signal assignment [42]. Robust quantum mechanical calculations allow for the identification and energy ranking of multiple low-energy conformers, providing a more comprehensive and accurate foundation for interpreting thermally averaged NMR experimental data. This ensures the critical importance of integrating experimental and computational approaches in NMR studies. While experimental measurements alone may result in misassignments due to unresolved averaging effects, DFT-based calculations offer a rigorous framework for deconvoluting overlapping signals, validating spectral assignments, and ensuring structural accuracy.

The ^13^C-NMR (TMS), ^14^N-NMR (NH_3_), and ^17^O-NMR (H_2_O) chemical shifts δ_C_ of VTB (global minimum structure) based on DFT mPW1W91/6-311++G(d,p) calculations in DMSO solvent are given on the structure of VTB in Figure S6. The Mulliken charges of the non-hydrogen atoms, which are calculated using the same level of theory, are displayed by color in this figure.

As shown in the results in Table 2 and the assignments in Figure S6, the aromatic ^13^C NMR chemical shifts of VTB are well separated from the aliphatic ones, with aromatic carbons appearing at δ_C_ > 90 ppm and aliphatic carbons at δC < 70 ppm. The most deshielded carbons (δ_C_ > 140 ppm) are located in the aromatic ring and are directly bonded to electronegative atoms such as nitrogen (N), oxygen (O), and fluorine (F). Additionally, the carbon atoms in VTB exhibit either positive (green) or negative (red) Mulliken charges, depending on their bonding environments. Generally, the more negative the Mulliken charge on a carbon atom, the more shielded it is; conversely, the more positive the charge, the more deshielded its chemical shift. For example, the most deshielded carbon atom (δC = 153.92 ppm) is the carbon in the quinazoline ring that is bonded to the –O–CH_2_– bridge.

**Figure 7:**
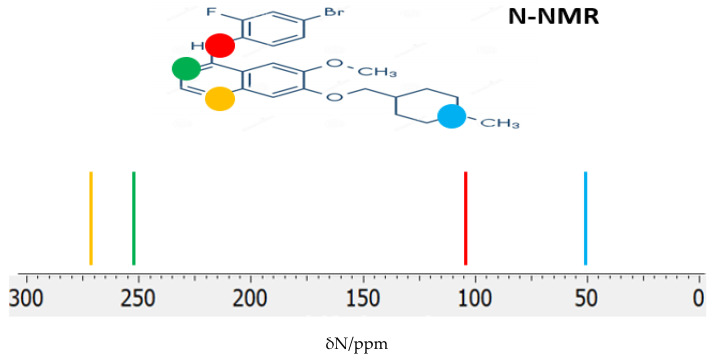
The ^14^N-NMR chemical shifts (δ_N_) of VTB were calculated using mPW1PW91/6-311++G(d,p) in DMSO (the global minimum structure). The ^14^N-NMR chemical shifts of aromatic (more deshielded) and aliphatic (more shielded) Ns are well separated.

The ^14^N NMR chemical shifts (δ_N_) provide valuable insights into the chemical environment and electronic structure of nitrogen atoms in pharmaceutical inhibitors. In VTB, the four nitrogen atoms occupy distinct chemical environments, leading to well-differentiated δ_N_ values in the calculated ^14^N NMR spectrum (Figure 7). For the global minimum structure, the predicted δN values are 274.15 ppm, 250.89 ppm, 110.27 ppm, and 53.04 ppm, corresponding to the nitrogen atoms in the quinazoline ring, the bridging secondary amine (-NH-), and the piperidine nitrogen, respectively. In these calculations, ammonia (NH_3_), with a shielding constant σ_N_ = 258.4 ppm, was used as the reference compound for determining the chemical shifts. These distinct δ_N_ values reflect the varying degrees of electron density and local chemical environments surrounding each nitrogen atom, consistent with their roles in the molecule’s aromatic and aliphatic regions.

### 2.3. UV–Vis Spectra of Low-Lying Vandetanib

**Figure 8:**
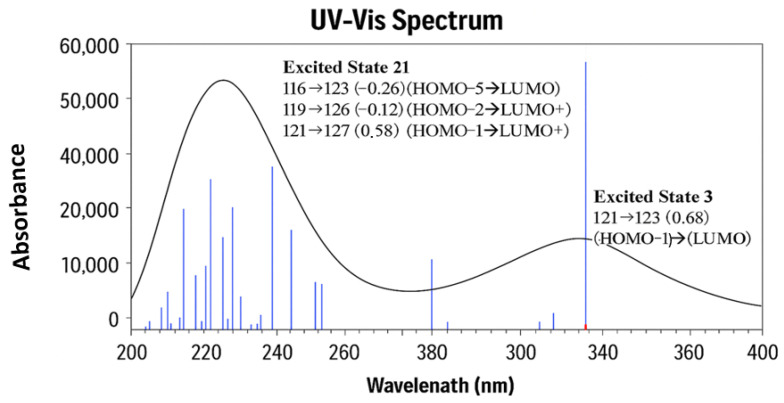
UV–vis calculated absorption spectrum of the global minimum structure of VTB in methanol solution in the range of 200–400 nm. Here molecular orbital 122 (MO122) is the highest occupied molecular orbital (HOMO), and molecular orbital 123 (MO123) is the lowest unoccupied molecular orbital (LUMO) for VTB.

**Figure 9:**
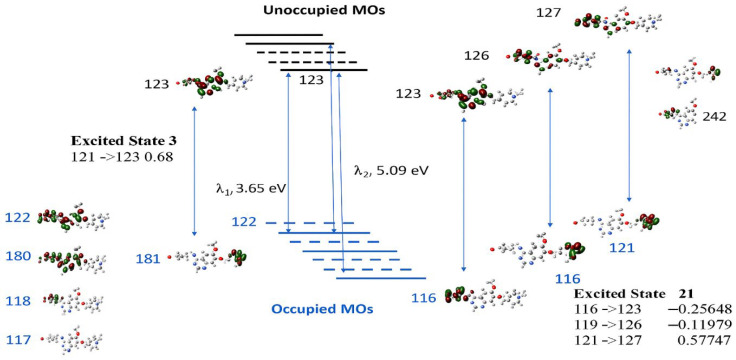
The excited states of the transitions and the related molecular orbitals (MOs). The number associated with the MO electron density distributions is the orbitals. Here, MO122 is the HOMO, and MO123 is the LUMO. The MOs involved in the transitions are presented by solid lines, and other MOs not involved in the above transitions are presented as dashed lines.

The UV–visible absorption spectrum of the global minimum conformer of VTB was calculated using the DFT B3PW91/6-311++G(d,p) method, incorporating methanol. As shown in Figure 8, the calculated UV–vis spectrum in the 200–400 nm range reveals two characteristic absorption bands that correspond to the key electronic transitions of the molecule’s aromatic (R1, R2, and R3) and heterocyclic (N-methyl piperidine (R4)) systems. The primary absorption band (λ_max_) is predicted at 339.65 nm, corresponding to an *n* → π* charge transfer (CT), predominantly localized on the N-methyl piperidine ring (R4) to the quinazoline and substituted aniline fragments (R1-R3). This primary band is attributed to a single dominant electronic excitation, as illustrated by the excitation contributions in Figure 9. Importantly, this calculated absorption is in excellent agreement with available experimental data, which report similar λ_max_ values of 339 nm [40], 328.44 nm in methanol [16,38], and 330.31 nm [17] in methanol mixtures. The absence of significant absorbance beyond 350 nm confirms that VTB lacks extended chromophores absorbing in the visible region, a finding consistent with its molecular structure, which contains a moderately conjugated aromatic system without extended delocalization sufficient to shift absorptions into the visible range.

The secondary absorption band (λ′_max_) is calculated at 243.48 nm, located in the deep UV region. Unlike the primary transition, which is dominated by a single strong transition, this band arises from a collection of several closely spaced electronic excitations, likely involving both π → π* and potential *n* → π* CT transitions associated with different regions of the molecule, including non-bonding electrons on heteroatoms. The composite nature of this band is clearly shown in the spectrum, where multiple transitions contribute to its intensity. This region also aligns well with experimental observations, where the corresponding absorptions were reported at 255 nm by Fei [40] and 249 nm by Al-Ghusn et al. [17].

It is noted that the transitions associated with these two bands do not contain HOMO (MO122)-LUMO (MO123) transitions; rather, they involve other orbitals. Although the calculated low-lying excited states, such as excited states 1 and 2, are dominated by transitions of HOMO (MO122) to LUMO+1 (MO124) at 487.71 nm and of HOMO (MO 122) to LUMO (MO 123) at 420.46 nm, the oscillator strength of the transitions are very small at f = 0.0017 and f = 0.0001, respectively. Figure 9 presents the collected information on the two major UV–vis transitions of VTB, calculated at the same DFT level of theory. A detailed analysis of these electronic transitions reveals the underlying orbital contributions that define the photophysical behavior of VTB. The primary absorption band, calculated at 339.65 nm (excited state 3, 3.65 eV, corresponding to a maximum oscillator strength f = 0.3878), indicates an intense, allowed transition. This excitation is primarily composed of transitions from HOMO–1 (MO 121) to LUMO (MO 123), with a major contribution (coefficient = 0.68), and a smaller contribution from HOMO-5 (MO 117) to LUMO 123 (0.10).

Although MO 122 is identified as the HOMO, the primary absorption arises predominantly from the HOMO–1 (MO 121) → LUMO (MO 123) transition rather than a direct HOMO → LUMO excitation. Both the HOMO and LUMO are primarily localized on the anilino-quinazoline rings (R1–R3), so a direct HOMO → LUMO transition would involve minimal spatial separation and therefore limited charge transfer (CT). In contrast, HOMO–1 (MO 121) is mainly localized on the N-methyl piperidine ring (R4), while the LUMO (MO 123) is centered on the quinazoline moiety (R2–R3). This spatial separation between the donor and acceptor orbitals facilitates a strong CT transition. Specifically, the HOMO–1 → LUMO excitation represents a redistribution of electron density from the aliphatic piperidine fragment to the aromatic quinazoline system, which enhances the CT character and contributes significantly to the intensity of the primary absorption band (see Figure 9).

The secondary absorption band, calculated at 243.48 nm (excited state 21, 5.09 eV, *f* = 0.2516), is more complex and arises from multiple orbital contributions. The dominant excitation is from HOMO–1 (MO 121) → LUMO+4 (MO 127), with a significant coefficient of 0.58, accompanied by smaller contributions from HOMO–6 (MO 116) → LUMO (MO 123) (−0.26), HOMO–3 (MO 119) → LUMO+3 (MO 126) (−0.12), and HOMO–1 (MO 121) → LUMO+5 (MO 128) (−0.10). As illustrated in Figure 9, these excitations involve transitions from orbitals significantly deeper than HOMO–1 (e.g., HOMO–6 and HOMO–3) to higher-energy unoccupied orbitals (LUMO+3 to LUMO+5). A common feature among these transitions is the spatial separation of the electron densities between the involved molecular orbital pairs, which facilitates effective charge transfer (CT). Similar to the primary absorption transition, the MOs involved in this band are localized in distinct molecular regions: the occupied orbitals are predominantly situated on the piperidine ring (R4) or the halogen-substituted phenyl ring (R1), while the unoccupied orbitals are localized on the quinazoline moiety (R2–R3). This clear donor–acceptor separation enables substantial intramolecular charge transfer upon excitation, contributing to the overall intensity and character of the secondary UV absorption band.

A comparative analysis of the UV–vis absorption spectra of several clinically relevant EGFR tyrosine kinase inhibitors (TKIs)—vandetanib, gefitinib, dacomitinib, and afatinib—reveals consistent spectral features that reflect their shared halogenated anilino-quinazoline core structure. As shown in Table 3, all compounds exhibit two characteristic absorption bands: a primary absorption band (λ_max_) in the UVA region (~330–343 nm) and a secondary band in the UVB region (~244–261 nm). Specifically, the obtained primary absorptions are centered around 339 nm for VTB, 332 nm for gefitinib [43], 343 nm for dacomitinib [44], and 340 nm for afatinib [45,46]. These transitions are typically dominated by π(*n*) → π* excitations involving the conjugated quinazoline and aniline π-systems, which are structurally conserved across the series. The similarity in these λ_max_ values suggests that the electronic transitions are governed largely by the extended π-conjugation and electron-withdrawing effects of the halogen substituents, which modulate the frontier molecular orbitals in a consistent manner.

The secondary UVB absorption bands, observed between 244 and 261 nm, are more variable in energy and likely arise from higher-energy π → π* or n → π* transitions involving non-bonding electrons on heteroatoms (e.g., nitrogen and oxygen) in different peripheral groups or side chains. The slight differences in λ′ₘₐₓ among the TKIs may reflect variations in the substituent electronics or conformational flexibility beyond the conserved core. This pattern of UV–vis absorption confirms that the halogenated anilino-quinazoline scaffold acts as a chromophoric unit responsible for consistent UVA absorption across EGFR-TKIs. This also explains the common occurrence of photosensitivity side effects in this drug class, as their strong UVA absorption allows them to be photoactivated under ambient light conditions, potentially generating reactive intermediates. The UVA absorption of VTB is driven by CT, while the UVB band arises from a combination of deeper electronic excitations within the molecular framework. These insights provide a mechanistic explanation for VTB’s photochemical reactivity under UVA light, contributing to its photosensitivity side effects. Understanding these photophysical similarities may guide the design of next-generation TKIs with reduced phototoxic risk while retaining EGFR-targeting efficacy.

The similarity in the absorption features across multiple conformers (Figure S5 in SM) indicates that VTB retains its electronic characteristics regardless of conformational differences, suggesting that its UV absorption is dominated by the aromatic and heterocyclic chromophores common to all conformers. The calculated λ_max_ and λ′_max_ values support the photochemical stability of VTB in the UVB and UVA regions, although the presence of absorption in the UVA range also correlates with its reported photosensitivity side effects, likely due to photoexcitation-induced reactive species generation.

From a pharmacological perspective, VTB’s conformational flexibility and photophysical properties may also relate to its dual-target inhibitory activity. The dominant low-energy conformers (Conformers 01 and 02, together accounting for over 90% of the conformational population) exhibit UV absorption features closely matching the experimental values. These conformers present the molecular geometry necessary for binding the ATP-binding site of EGFR, a well-established mechanism of action. Additionally, computational studies have suggested that VTB could interact with viral proteins, such as the SARS-CoV-2 main protease (Mpro) or spike protein, through flexible side chains adapting to distinct binding pockets. The presence of multiple low-lying conformers provides a structural basis for such adaptability, enabling VTB to act as a dual inhibitor of both human tyrosine kinases and viral enzymes.

Vandetanib is known to cause photosensitivity reactions in some patients [47], and its UV–vis absorption spectrum provides a molecular basis for this side effect. The spectrum of VTB shows two prominent absorption bands: one in the UVB region (~250 nm) and another in the UVA region (~340 nm) [48]. The 250 nm band corresponds to high-energy π(*n*) → π* transitions within the aromatic and heterocyclic systems of the quinazoline and aniline moieties. More importantly, the absorption at 340 nm falls within the UVA region (320–400 nm), which overlaps with the wavelengths present in sunlight that penetrate the Earth’s atmosphere and human skin [47].

This suggests that upon UVA exposure, VTB can absorb solar radiation and enter excited electronic states, potentially leading to the generation of reactive oxygen species (ROS) or photoinduced chemical reactions in the skin [47]. These photochemical processes can damage cellular components and trigger inflammatory responses, resulting in the photosensitivity symptoms reported in clinical use. Therefore, the UV–vis absorption profile of VTB provides a mechanistic explanation for its photosensitivity side effect, underscoring the importance of photoprotection measures, such as avoiding sun exposure or using broad-spectrum sunscreens, during treatment.

## 3. Materials and Methods

The chemical, DFT-optimized, and crystal structures of VTB are shown in Figure 2. All quantum mechanical calculations, which include geometry optimization, NMR, and UV–vis spectral simulations, were performed using density functional theory (DFT) and time-dependent DFT (TD-DFT) methods, as implemented in the Gaussian 16 software package [48].

To locate a global minimum and several low-energy minima, the PrefQMCon method developed by Wang and Vasilyev was applied. This method generated 300 random conformers by rotating six key single bonds of VTB: N(21)–C(22), N(21)–C(20), O(9)–C(10), O(9)–C(8), C(7)–C(8), and C(11)–O(12) (see Figure S1 in the Supplementary Material). Each structure was optimized in dimethyl sulfoxide (DMSO) using the B3LYP/6-311++G(d,p) level of theory, with a linear-response polarizable continuum model (LR-PCM) to simulate solvent effects. The PrefQMCon method then performed clustering of the resulting optimized structures based on their geometries and energies (see ref. [32] for details). As a result, the global minimum structure and several low-energy minima were identified for further calculations.

For NMR calculations, the low-energy conformers were re-optimized using the mPW1PW91/6-311++G(d,p) level of theory in DMSO, following the recommendations of Dickson et al. [34] for reliable biomolecular NMR shift prediction. For UV–vis spectral simulations, time-dependent density functional theory (TD-DFT) calculations were performed at the B3LYP/6-311++G(d,p) level in methanol, based on re-optimized conformers. The choice of B3LYP is supported by benchmarking studies on 4-anilinoquinazoline–based EGFR inhibitors, such as AG-1478 [35] and PD153035 [20], which have demonstrated that this level of theory provides reliable reproduction of experimental absorption spectra. The solvent in both NMR and UV–vis calculations was modeled as (DMSO, dielectric constant ε = 46.82 at 20 °C) using the Gaussian default polarizable continuum model (PCM). For UV–vis simulations, singlet transitions within the lowest 30 singlet excited states were considered. Convergence criteria were set to the Gaussian program defaults, with 128 cycles of self-consistent field (SCF), density convergence of 10^−8^, and energy convergence of 10^−6^ E_h_.

## 4. Conclusions

This study provides detailed structural and UV–vis spectral insights into vandetanib’s photosensitivity using molecular-level density functional theory (DFT) analyses. Vandetanib, an FDA-approved EGFR tyrosine kinase inhibitor for medullary thyroid cancer (MTC), has recently emerged as a potential dual inhibitor targeting SARS-CoV-2 main protease (Mpro), highlighting its broad therapeutic relevance.

Robust DFT calculations were carried out on low-energy conformers of vandetanib in solution, validated against available experimental data, including crystal structures and NMR measurements. This integrative approach enabled an improved interpretation of thermally averaged NMR data and emphasized the value of computational support in resolving conformational and spectroscopic ambiguities. Analysis of the UV–vis absorption spectrum identified a key electronic transition (HOMO–1 → LUMO) involving charge transfer from the N-methyl piperidine ring to the quinazoline core, with pronounced absorption in the UVA region (~339 nm). This behavior aligns with clinically observed phototoxic effects and is further linked to the known metabolic reactivity of the same piperidine moiety [16].

While this work focused on the ground and singlet excited states to explore vandetanib’s photophysical properties, we acknowledge that its photosensitivity likely involves additional pathways. In particular, reactive oxygen species (ROS) generation and intersystem crossing (ISC) to triplet states represent important facets of phototoxicity. Future work should incorporate time-dependent DFT (TD-DFT) calculations on triplet excited states, evaluation of singlet–triplet energy gaps, and spin–orbit coupling to provide a more complete mechanistic understanding of vandetanib’s photoreactivity.

In summary, this study highlights how vandetanib’s conformational flexibility, electronic structure, and photophysical properties contribute to both its dual biological activity and photosensitive risk profile. These findings support further development of predictive models to optimize the safety and expand the therapeutic scope of this important drug class.

## Figures and Tables

**Table 1:** Comparison of selected properties of three low-energy VTB conformers *.

Parameters	Vandetanib Low-Lying Conformers			
	Van1	Van2	Van3	Crystal ^#^
**R_1_ (Ả)**	8.34	8.34	8.34	8.38
**R_2_ (Ả)**	8.18	8.18	8.19	8.18
**R_3_ (Ả)**	8.42	8.42	8.41	8.37
**R_4_ (Ả)**	9.01	9.01	9.01	9.06
**<R^2^> (a.u.)**	108,955.76	99,622.38	49,763.67	701,592.17
***μ* (D)**	5.58	4.99	5.97	7.66
***α* (a.u)**	461.05	461.07	459.48	509.38
***E*_h_ + ZPE (a.u.)**	−3896.0036	−3896.0027	−3895.9993	−3896.5936

* Using mPW1PW91/6-311++G(d,p) level of theory in DMSO. ^#^ Recent crystal structure [11].

**Table 2:** Comparison of the DFT calculated proton NMR chemical shifts δ_H_ of the global minimum structure of Vandetanib (Van1) in DMSO with measurements (ppm) *.

Sites	Cals		Expt		
	Vx	Vx′	Al-Ghusn et al. [17]	Brocklesby et al. ^#^ [41]	Fei [40] ^
	B3	mPW1	Δδ_C_ (mPW1)	Δδ_C_ (mPW1)	Δδ_C_ (mPW1)
C1	26.63	25.10	−2.33	−3.58	−3.90
C2	29.86	26.73	0.90	−1.95	−8.27
C3	35.10	32.72	0.02	−2.20	−5.28
C4	42.10	41.09	−4.59	−5.89	1.09
C5	52.63	50.17	−2.71	0.85	3.17
C6	53.24	51.01	−2.10	−4.68	−3.99
C7	54.36	51.57	−2.27	−4.12	−4.43
C8	71.80	69.41	−1.45	−10.45	−3.59
C9	98.06	96.75	−4.35	−6.07	−5.25
C10	108.07	106.26	0.55	−1.26	1.26
C11	108.25	106.93	−1.37	−1.98	−1.07
C12	120.46	117.80	2.48	3.06	0.80
C13	127.54	120.40	7.81	2.75	0.40
C14	128.98	127.43	2.13	6.22	1.43
C15	129.32	127.56	1.36	−0.12	−2.44
C16	136.50	129.20	6.49	−2.12	−2.80
C17	147.30	146.28	−0.11	−6.76	−0.72
C18	153.28	149.71	5.71	−5.52	−0.29
C19	154.56	152.24	1.18	−3.75	−0.76
C20	155.03	152.63	0.77	−4.45	−3.37
C21	155.91	152.67	−0.50	−6.19	−5.33
C22	160.22	153.92	2.87	−9.77	−4.71
**RMSD**			**3.15**	**5.01**	**3.57**

* For full table, see Table S5 in SM. DFT calculations using the Vxc/6-311++G(d,p) level of theory. Vx = B3 for B3PW91 (see RMDS ^$^); Vx′ = m for mPW1PW91 (See RMSD **^%^**). The carbon chemical shifts for B3 are calculated for the global minimum structure. Here, Δδ_C_ = δ_C_(cal) − δ_C_(expt). ^#^ The measured digital chemical shift values of VTB ^13^C-NMR of Brocklesby et al. [41] missed the degenerated bands due to the overlap of the C-signals [17]. Here, the corrected ^13^C-NMR chemical shifts of Brocklesby et al. [41] is used. ^ This reference only gives the DEPTQ- and DEPTQ135-measured ^13^C-NMR spectra of VTB [40] without digital chemical shift values. The numbers in the table are estimated chemical shifts from the DEPTQ and DEPTQ135 spectra.

**Table 3:** UV–vis maximum absorption positions of selected anilino quinazoline-based kinase inhibitors (nm).

EGFR-TKI	λ_max_ (nm)	Ref.
Vandetanib	244, 339	Present
Gefitinib	250, 332	[43]
Dacomitinib	261, 343 ^#^	[44]
Afatinib	246, 340	[45,46]

^#^ In DMSO.

## Data Availability

The original contributions presented in this study are included in the article/Supplementary Material. Further inquiries can be directed to the corresponding author.

## Supplementary Materials

The following supporting information can be downloaded at: https://www.mdpi.com/article/10.3390/ph18091297/s1.
