# Supplementary material for: DFT Structural and UV–Vis Spectral Insights into Photosensitivity of Vandetanib: A Dual EGFR/SARS-CoV-2 Mpro Inhibitor
Source: Pharmaceuticals (Basel). 2025 Aug 29;18(9):1297. doi: 10.3390/ph18091297 (PMC12472933; doi:10.3390/ph18091297)
Supplement: Supplementary file 1 [file pharmaceuticals-18-01297-s001.zip › pharmaceuticals-3803255-supplementary.pdf]

DFT Structural and UV–Vis Spectral Insights into Photosensitivity of Vandetanib: A Dual EGFR/SARS-CoV-2 Mpro Inhibitor

Feng Wang [1,*] and Vladislav Vasilyev [2]

[1] School of Science, Computing and Emerging Technologies, Swinburne University of Technology, Melbourne, VIC 3122, Australia
[2] National Computational Infrastructure, Australian National University, Canberra, ACT 0200, Australia; vvv900@nci.org.au

Supplementary Materials

* Correspondence: fwang@swin.edu.au

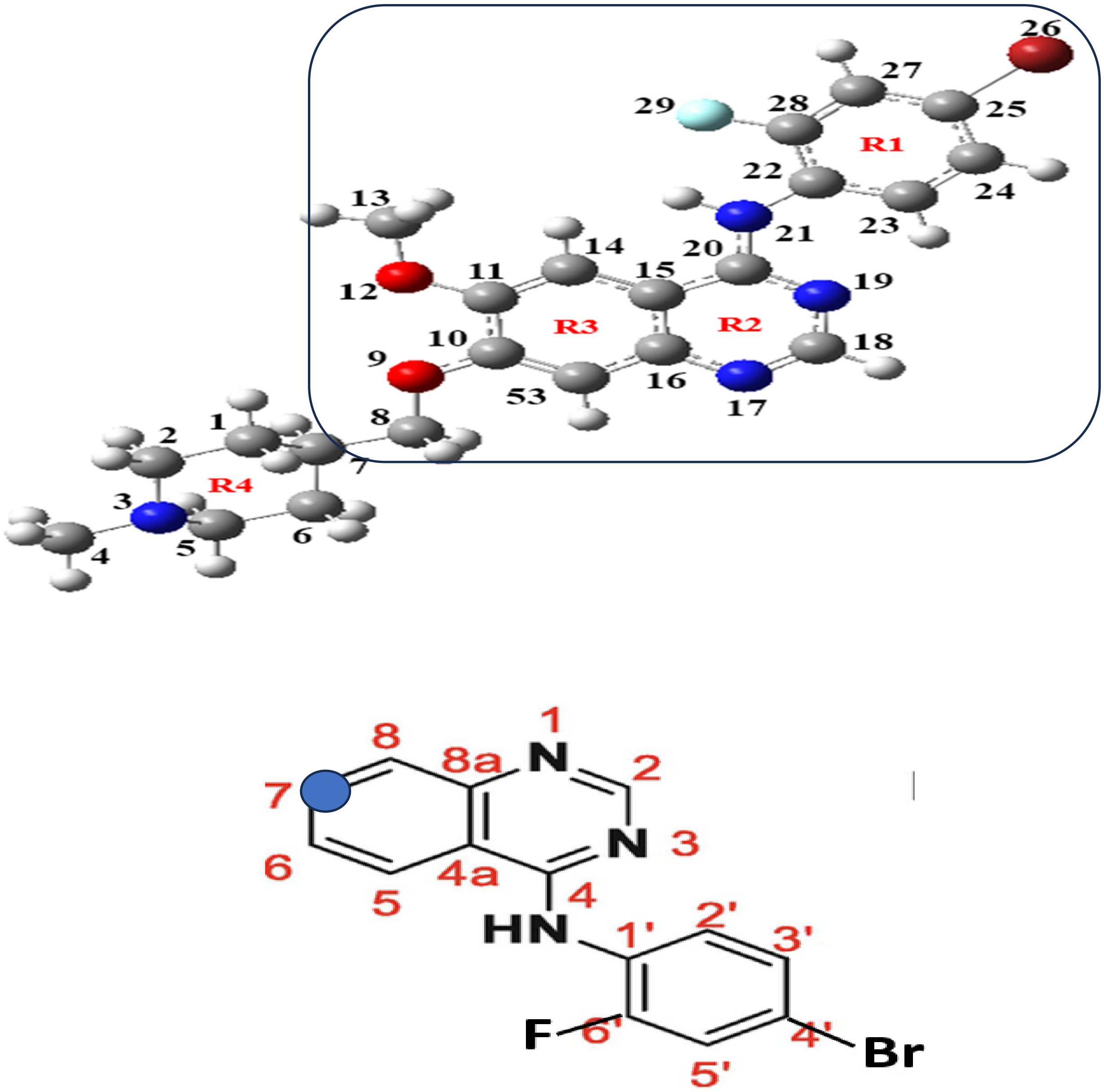

Figure S1 The optimized ground electronic state structure of Vandetanib using B3PW91/6-311++G(d,p) in DMSO solvent using the PCM solvent model (above). The quinazoline skeleton (below) where the potent C7 position is marked on the skeleton The nomenclature of quinazoline fragment (the fragment in the highlight). Note that the numbers in Vandetanib are the Gaussian labelling of the atoms with the definition of R_1, R_2 and R_3 and R_4 perimeters for three aromatic and piperidine rings, respectively. The nomenclature in the quinazoline fragment is the IUPAC labelling.

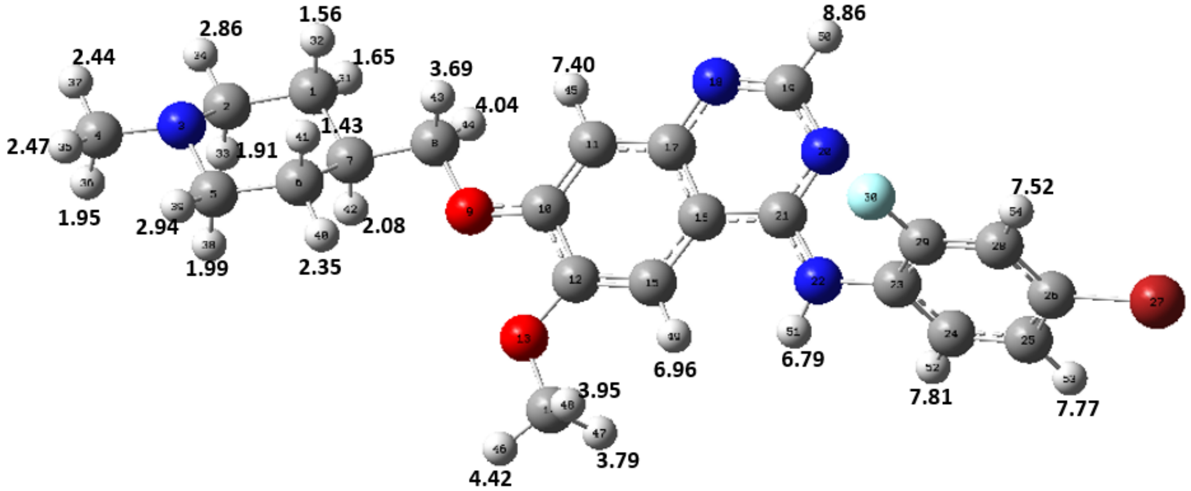

Figure S2 The calculated H-NMR chemical shifts δ_H of Vandetanib using B3PW91/6-311++G(d,p) in DMSO solvent (above), and the calculated H-NMR spectra of VAN (below). Noted that DMVAN lacks of two protons on the methyl group (i.e. -$C_{(4)}H_3$ in the structure) and the largest chemical shift bridge -HN- proton is ca 10 ppm [Brocklesby, 2017 #418].

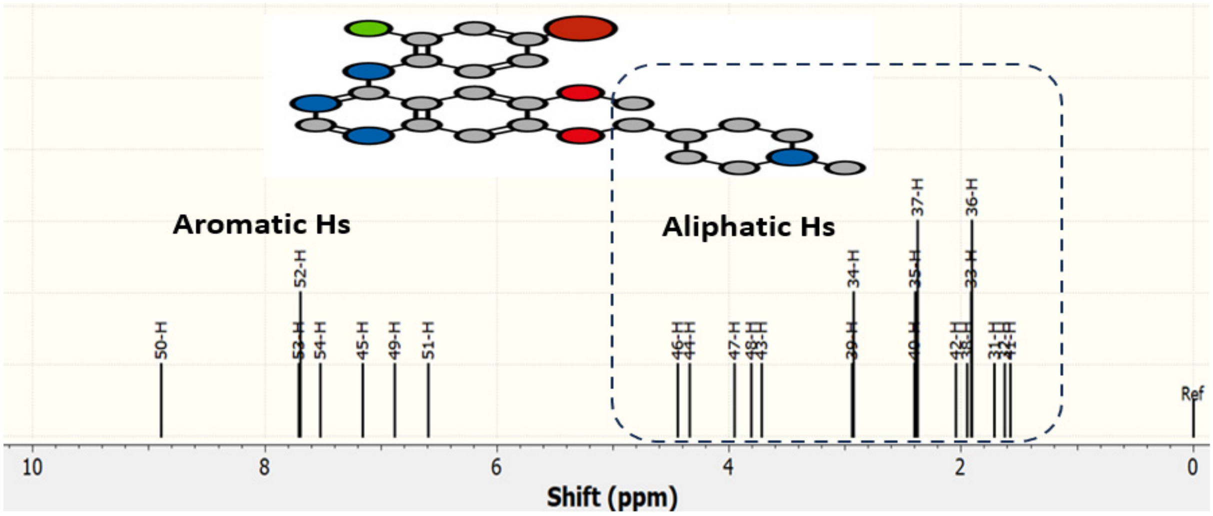

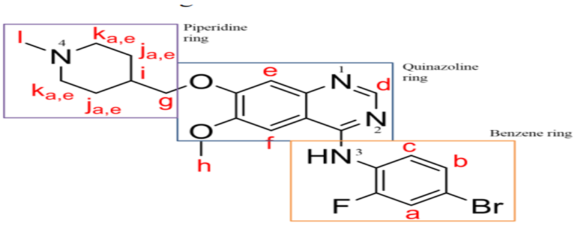

Fig. 2.3 Structure of vandetanib.

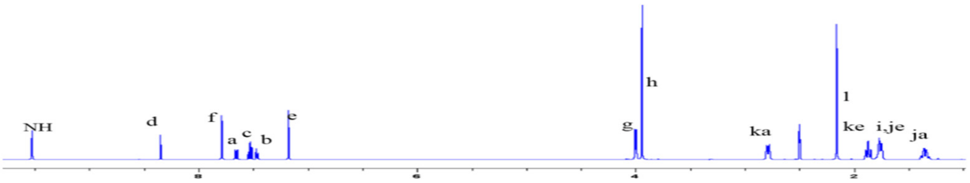

Fig. 2.4 ^{1}H NMR spectrum of vandetanib.

Figure S3 (a) The proton-NMR chemical shifts of VTB, (b) the aromatic proton-NMR (7 Hs) chemical shifts, and (c) the aliphatic proton-NMR (17 Hs) chemical shifts of VTB (Van1). All are calculated using mPW1PW91/6-311++G(d,p) in DSMO solvent.

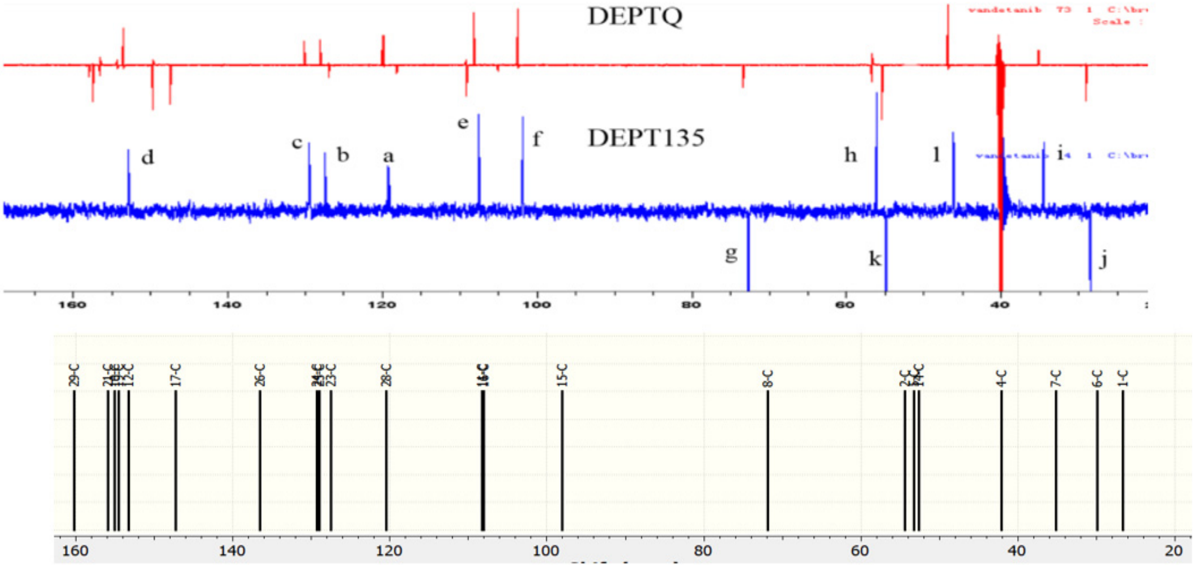

Figure S4 Comparision of the calculated and measured [1] C-NMR spectrum in DMSO solvent.

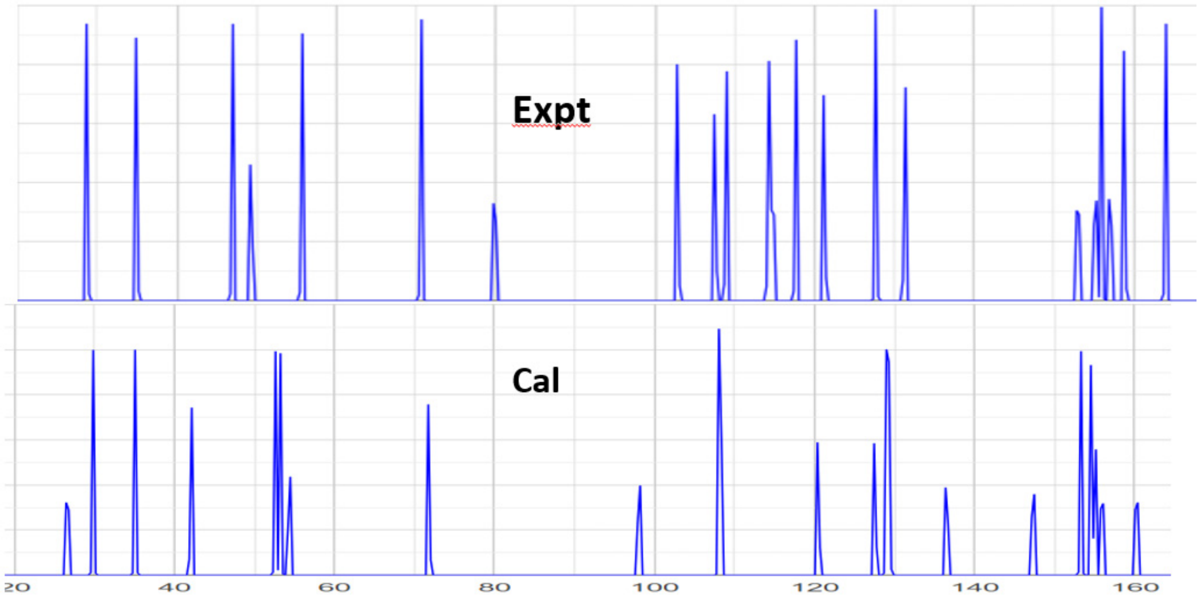

Figure S5 Comparision of the measured and calculated ^{13}C-NMR of vandetanib in DMSO solvent.

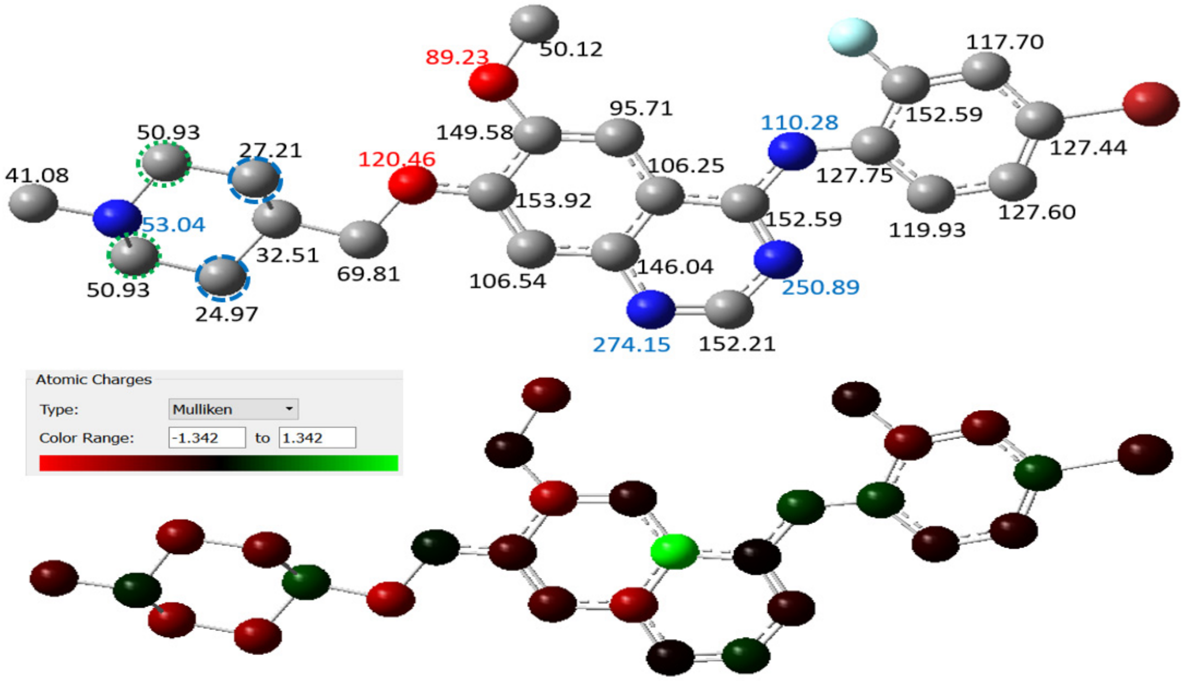

Figure S6 The calculated and assignment of ^{13}C-NMR (TMS), ^{14}N-NMR (NH_3) and ^{17}O-NMR (H_2O) chemical shifts δ_C of VTB based on DFT mPW1W91/6-311++G(d,p) calculations in DMSO solvent (above). The Mulliken charge of the non-hydrogen atoms by colour.

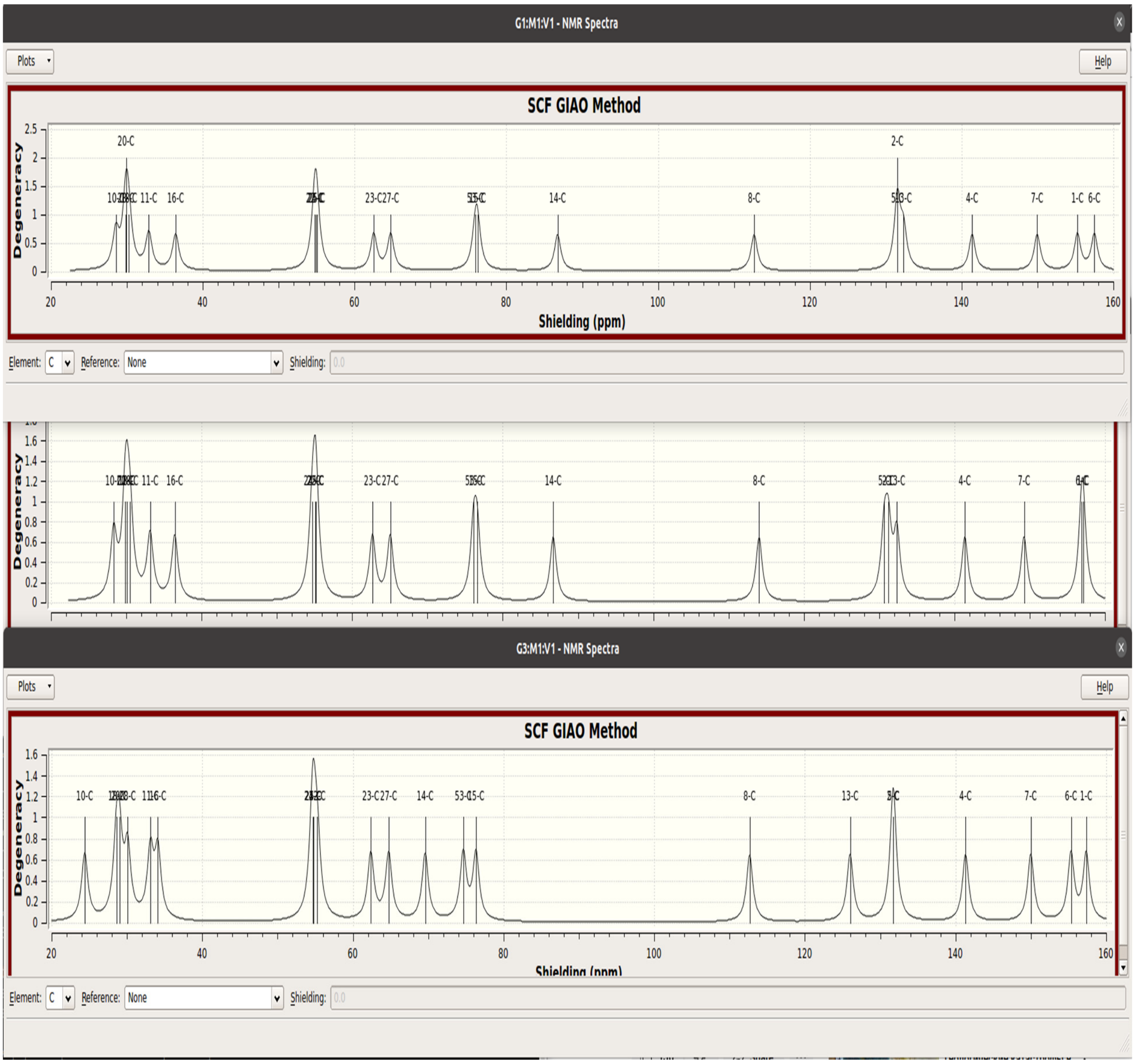

Figure S7 The calculated ^{13}C-NMR chemical shifts δ_H of three low-lying Vandetanib conformers using mPW1PW91/6-311++G(d,p) in DMSO solvent. From the top to bottom are Van1 (global minimum structure), Van2 and Van3.

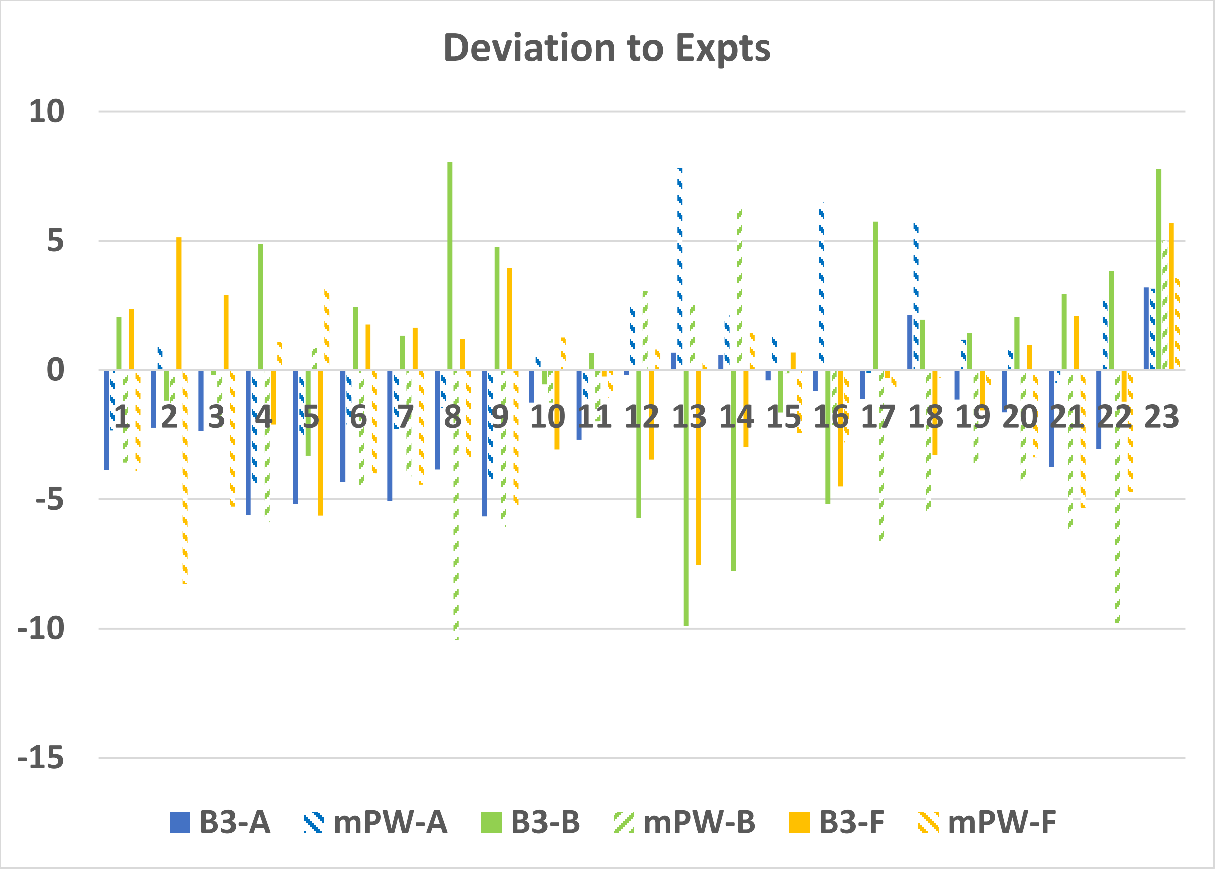

Figure S8 Comparison of deviations of the δC calculated using two DFT Vx's, Vx =B3 (solid) and Vx=mPW91 (pattern), B3PW91/6-311++G(d,p) and mPW1PW91/6-311++G(d,p), with three different experimental measurements of Al-Ghusn et al. [32] (A, blue); of Brocklesby et al. [31] (B, green) and of Fei [30] (orange, C).

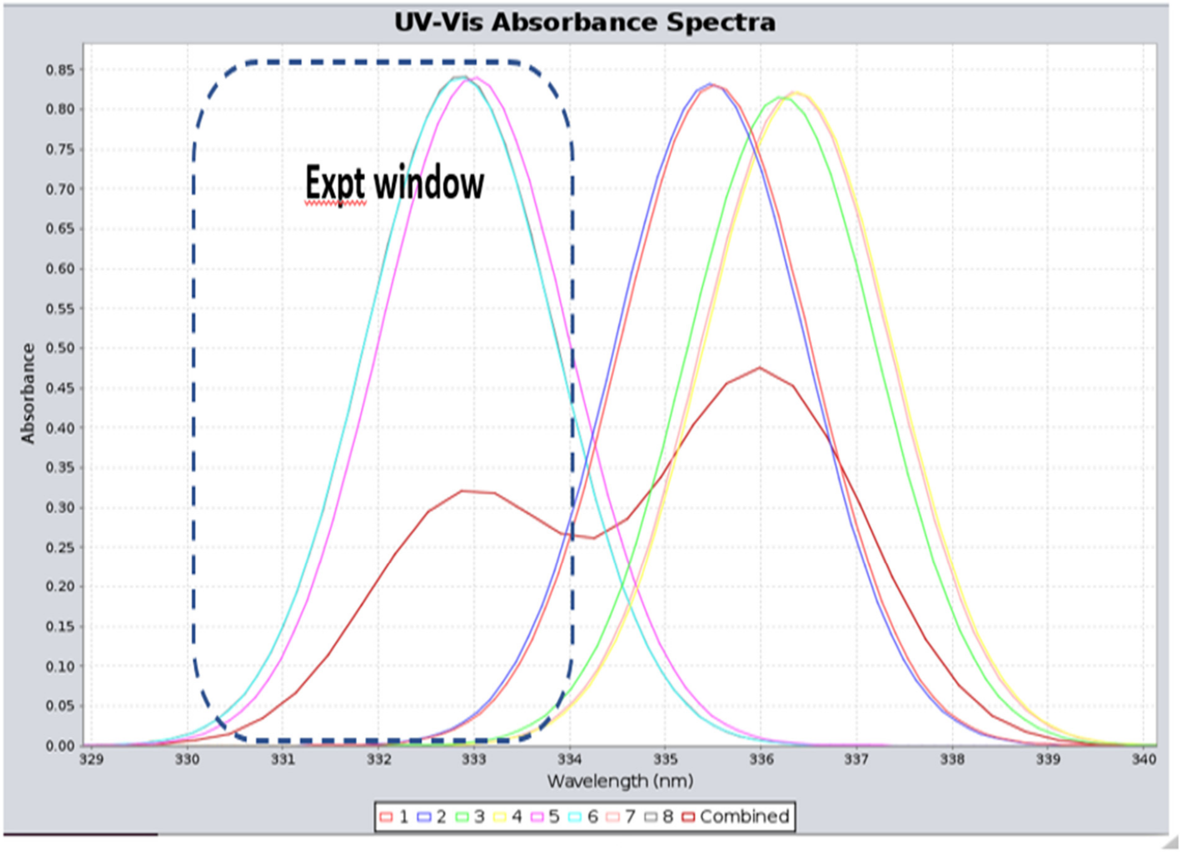

Figure S9 UV-Vis spectra of the 8th low-lying Vandetanib conformers in methanol solvent calculated using B3PW91/6-311++G(d,p) model of theory.

Table S1. The conformers of low-lying Vandetanib.*

Clusters #	Total Energy (E_h)	E_{SE} (kcal/mol)	μ (Debye)	Weight%
1	-3896.0689652	0.000	5.6805	62.898
2	-3896.0682304	0.461	5.0943	28.492
3	-3896.0669054	1.293	4.2040	6.832
4	-3896.0644363	2.842	5.9718	0.477
5	-3896.0640799	3.066	6.0406	0.325
6	-3896.0638363	3.218	5.5077	0.250
7	-3896.0637106	3.297	5.2542	0.218
8	-3896.0630335	3.722	5.6484	0.105
9	-3896.0628787	3.819	6.6941	0.089
10	-3896.0628504	3.837	6.6939	0.086
11	-3896.0628437	3.841	6.6862	0.086
12	-3896.0620991	4.308	4.2821	0.038
13	-3896.0619888	4.378	6.7827	0.034
14	-3896.0619626	4.394	5.9012	0.033
15	-3896.0617386	4.535	4.8093	0.026
16	-3896.0597192	5.802	5.9916	0.003
17	-3896.0595058	5.936	6.2764	0.002
18	-3896.0590077	6.248	5.9629	0.001
19	-3896.0586257	6.488	5.7861	0.001
20	-3896.0576526	7.099	5.4695	0.000
21	-3896.0569192	7.559	4.1922	0.000
22	-3896.0566012	7.758	5.3845	0.000

*DFT based B3LYP/6-311++G(d,p) level of theory in dimethyl sulfoxide (DMSO). Canonical partition function at 293.0 K with a strain energy cut off at 7.758 kcal/mol above the global minimum structure [2].

Table S2. Comparison of selected optimized geometrical parameters of Vandetanib and its crystal structure [2].*

**Parameters (Å, °)**	Vandetanib		$\Delta_{(Cal-Expt)}$
	**Full Opt**	**Crystal**	
R_1	8.3538	8.35378	2E-05
R_2	8.20958	8.21052	-0.00094
R_3	8.44364	8.44315	0.00049
R_4	9.06242	9.06373	-0.00131
$\langle R^2 \rangle$ (a.u.)	701592.171	701592.171	0
μ (D)	7.989476	7.664091	0.325385
α (a.u)	454.91	509.38	-54.47
E_h (a.u.)	-3896.59685	-3896.5948	-0.00209
ZPE (kcal.mol^{-1})	271.8079	271.522388	0.285513
E_h + ZPE (a.u.)	-3896.59685	-3896.1621	-0.43479
$\varepsilon_{HOMO-LUMO}$ (eV)	0.16208	0.16139	0.00069

*Using B3LYP/6-311++G(d,p) basis set in DMSO solvent.

Table S3 Comparison of DFT calculated ^{1}H-NMR chemical shifts of three low-lying conformers of VTB with available experimental measurements.*

δ_H	DFT-Cal				Expt			
	**Van1**	**Van2**	**Van3**	**Van weight**	**Smith** [3]	**Brocklesby** [4]	**Fei [1]**	**Al-Ghusn** [5]
H1	1.336	1.595	1.328	1.410	2.43	1.44	1.3518	1.35
H2	1.425	1.621	1.433	1.483	2.43	1.44	1.3528	1.35
H3	1.647	1.689	1.641	1.659	2.64	1.85	1.7634	1.76
H4	1.936	1.932	1.957	1.937	2.64	1.85	1.7634	1.76
H5	1.967	1.963	1.957	1.965	2.64	1.97	1.7724	1.76
H6	2.009	2.024	1.989	2.013	2.64	1.97	1.8728	1.86
H7	2.041	2.063	2.052	2.049	2.73	1.97	1.8728	1.86
H8	2.274	2.074	2.277	2.216	2.73	2.32	2.1612	2.16
H9	2.469	2.461	2.444	2.465	3.82	2.32	2.1612	2.16
H10	2.469	2.522	2.444	2.483	3.82	2.32	2.1612	2.16
H11	2.876	2.916	2.874	2.888	3.82	2.85	2.7903	2.79
H12	2.898	2.959	2.897	2.916	3.82	2.85	2.7903	2.79
H13	3.717	3.972	3.530	3.778	4.02	3.98	3.9743	3.95
H14	3.919	3.988	3.692	3.924	4.02	3.98	3.9743	3.95
H15	3.919	4.078	4.074	3.976	4.02	3.98	3.9437	3.95
H16	4.006	4.086	4.120	4.038	4.26	4.03	4.0009	4.01
H17	4.426	4.464	4.460	4.440	4.26	4.03	4.0009	4.01
H18	7.164	7.217	7.467	7.201	7.13	6.99	7.1778	7.18
H19	7.368	7.348	7.643	7.382	7.29	7.21	7.4643^	7.46
H20	7.606	7.597	7.660	7.608	7.49	7.26	7.4643	7.53
H21	7.638	7.624	7.876	7.651	7.61	7.34	7.5304	7.66
H22	7.768	7.806	7.916	7.790	7.98	7.36	7.6566	7.80
H23	8.926	8.875	8.898	8.910	8.66	8.51	7.7893$^{\&}$	8.36
H24	9.874	9.956	9.914	9.901	10.62$^{\$}$	8.68	9.5255	9.56
**RMSD$^{\#}$**	**0.29$^{\%}$**	**0.30$^{\%}$**	**0.33$^{\%}$**	**0.29$^{\%}$**	**0.71**	**0.32**	**0.29**	**0.21**

*DFT mPW1PW91/6-311++G(d,p) level of theory in DMSO solvent.

$^{\$}$If it is a range, the average values are taken.

^This aromatic proton chemical shift is missing in the measurement [1]. This value is taken as the previous one.

$^{\&}$This second last carbon chemical shift of this measurement seems too low, as all other measurements of this chemical shift are above 8 ppm, including the this value from the patent [6], which gives 8.34 ppm.

$^{\#}$Root mean square deviation (RMSD).

$^{\%}$The RMSDs of the proton NMR chemical shifts of VTB conformers are with respect to the measurement of Fei [1] as it is the most accurate measurement so far.

Table S4 Comparison of the DFT calculated aliphatic δH (17 Hs) of Vandetanib in DMSO solvent with measurements (ppm)*.

**Aromatic**	**Cal**	**Expt**			
**δ_H**		**Smith** [3]	**Brocklesby** [4]	**Fei**[36]	**Al-Ghusn [5]**
H1'	1.574	2.43	1.44	1.3518	1.35
H2'	1.574	2.43	1.44	1.3518	1.35
H3'	1.574	2.64	1.85	1.7634	1.76
H4'	1.625	2.64	1.85	1.7634	1.76
H5'	1.625	2.64	1.97	1.7724	1.76
H6'	1.625	2.64	1.97	1.8728	1.86
H7'	1.711	2.73	1.97	1.8728	1.86
H8'	1.711	2.73	2.32	2.1612	2.16
H9'	1.922	3.82	2.32	2.1612	2.16
H10'	1.922	3.82	2.32	2.1612	2.16
H11'	2.044	3.82	2.85	2.7903	2.79
H12'	2.392	3.82	2.85	2.7903	2.79
H13'	2.934	4.02	3.98	3.9437	3.95
H14'	3.716	4.02	3.98	3.9437	3.95
H15'	3.807	4.02	3.98	3.9437	3.95
H16'	3.954	4.26	4.03	4.0009	4.01
H17'	4.340	4.26	4.03	4.0009	4.01
**RMSD#**		**1.13**	**0.44**	**0.36**	**0.21**

*The chemical shift range is averaged. For nth degeneracy, takes the same value n times.

Table S5 Comparison of the ^{13}C-NMR chemical shifts of Vandetanib (C22H24BrFN4O2) in DMSO solvent (ppm)*

δc	Vx(B3)	Vx(m)	Al-Ghusn [5]	$\Delta\delta_C$ (B3)	$\Delta\delta_C$ (m)	CorrRef [4]	$\Delta\delta_C$ (B3)	$\Delta\delta_C$ (m)	Fei [1]^	$\Delta\delta_C$ (B3)	$\Delta\delta_C$ (m)
C1	26.63	25.101	28.96	-1.33	-2.33	28.68	2.05	-3.58	29	2.37	-3.90
C2	29.86	26.728	28.96	1.9	0.9	28.68	-1.18	-1.95	35	5.14	-8.27
C3	35.10	32.717	35.08	1.02	0.02	34.92	-0.18	-2.20	38	2.90	-5.28
C4	42.10	41.092	55.34	-3.59	-4.59	46.98	4.88	-5.89	40	-2.10	1.09
C5	52.63	50.165	55.34	-1.71	-2.71	49.32	-3.31	0.85	47	-5.63	3.17
C6	53.24	51.014	56.63	-1.1	-2.1	55.69	2.45	-4.68	55	1.76	-3.99
C7	54.36	51.568	73.25	-1.27	-2.27	55.69	1.33	-4.12	56	1.64	-4.43
C8	71.80	69.411	102.41	-0.45	-1.45	79.86	8.06	-10.45	73	1.20	-3.59
C9	98.06	96.746	109.02	-3.35	-4.35	102.82	4.76	-6.07	102	3.94	-5.25
C10	108.07	106.26	108.09	0.98	0.55	107.52	-0.55	-1.26	105	-3.07	1.26
C11	108.25	106.93	109.02	0.23	-1.37	108.91	0.66	-1.98	108	-0.25	-1.07
C12	120.46	117.8	117.98	3.48	2.48	114.74	-5.72	3.06	117	-3.46	0.80
C13	127.54	120.4	119.73	8.81	7.81	117.65	-9.89	2.75	120	-7.54	0.40
C14	128.98	127.43	126.85	3.13	2.13	121.21	-7.77	6.22	126	-2.98	1.43
C15	129.32	127.56	127.96	2.36	1.36	127.68	-1.64	-0.12	130	0.68	-2.44
C16	136.50	129.2	130.01	7.49	6.49	131.32	-5.18	-2.12	132	-4.50	-2.80
C17	147.30	146.28	147.41	0.89	-0.11	153.04	5.74	-6.76	147	-0.30	-0.72
C18	153.28	149.71	149.57	4.71	5.71	155.23	1.95	-5.52	150	-3.28	-0.29
C19	154.56	152.24	153.38	2.18	1.18	155.99	1.43	-3.75	153	-1.56	-0.76
C20	155.03	152.63	154.26	1.77	0.77	157.08	2.05	-4.45	156	0.97	-3.37
C21	155.91	152.67	156.41	0.5	-0.5	158.86	2.95	-6.19	158	2.09	-5.33
C22	160.22	153.92	157.35	3.87	2.87	164.06	3.84	-9.77	159	-1.22	-4.71
**RMSD**				**3.33**	**3.31**		**7.78**	**5.01**		**5.70**	**3.57**

*DFT calculations using the Vxc/6-311++G(d,p) level of theory. Vx = B3 for B3PW91 and Vx=m for mPW1PW91. The carbon chemical shifts for B3 are the calculated for the global minimum structure, but for m are the weighted carbon chemical shifts of three low-lying conformers of Vandetanib. Here $\Delta\delta_C = \delta_C(cal) - \delta_C(expt)$.

#The measured digital chemical shift values of VTB ^{13}C-NMR of Brocklesby et al [4] missed the degenerated bands due to overlap of the C-signals [5]. Here Corr[4] is the corrected ^{13}C-NMR chemical shifts of Brocklesby et al [4], refer to Al-Ghusn et al [5].

^This reference only gives the DEPTQ and DEPTQ135 measured ^{13}C-NMR spectra of VTB [1] without digital chemical shift values. The numbers in the table are estimated chemical shifts from the DEPTQ and DEPTQ135 spectra.

Table S6 The optimized structure of Vandetanib (global minimum) using B3PW91/6-311G(d,p) in DMSO solvent.

6 -25.58777898 28.45776877 48.32238752

6 -25.67717605 30.72731213 50.09200560

7 -23.14847030 31.66090859 50.66550821

6 -23.25151880 33.92027398 52.21904777

6 -21.61908498 29.69806832 51.85233589

6 -21.36946600 27.38577539 50.15599689

6 -23.96540402 26.33695347 49.43639799

6 -23.80460895 24.14306816 47.59864006

8 -22.49922675 22.10187049 48.80647593

6 -22.13113391 19.95831807 47.49749482

6 -22.91057991 19.54477476 45.05114639

6 -20.79597310 18.01039549 48.83428464

8 -20.08526517 18.62123237 51.20433531

6 -18.74108466 16.78317440 52.63366262

6 -20.32529607 15.73644872 47.67026000

6 -21.14792149 15.28888086 45.16440953

6 -22.43123829 17.21704914 43.83284511

7 -23.20605424 16.91145623 41.38895685

6 -22.69084612 14.71563750 40.36035959

7 -21.53968235 12.71893292 41.43463189

6 -20.78495706 12.98135507 43.80618391

7 -19.63105243 10.96427093 44.95239852

6 -19.16390991 8.63253667 43.76292776

6 -20.16618719 6.39180353 44.72381736

6 -19.63328091 4.06606515 43.62405247

6 -18.09477998 3.98989857 41.49893802

35 -17.37383705 0.83235611 39.93530322

6 -17.04939276 6.17819303 40.49087750

6 -17.58246337 8.45437972 41.65988600

9 -16.47470531 10.55209602 40.75721591

1 -27.51513342 27.78989958 47.97089141

1 -24.80045771 29.05332396 46.49890663

1 -26.68699936 30.19518591 51.84987707

1 -26.75672918 32.26088910 49.21948169

1 -21.34052303 34.63343666 52.55065188

1 -24.14573545 33.58042878 54.07745804

1 -24.33137616 35.39190742 51.25005696

1 -22.44790784 29.12549365 53.69062621

1 -19.74907214 30.48441196 52.25683582

1 -20.24783111 25.94273015 51.12051199

1 -20.34390794 27.91443388 48.43209752

1 -24.89904154 25.64746001 51.15892558

1 -22.77383201 24.69714685 45.88638892

1 -25.70008204 23.50938200 47.05119963

1 -23.90183715 20.98375336 43.98952221

1 -18.36596765 17.65628815 54.45788613

1 -19.87644487 15.07573085 52.90597233

1 -16.94990208 16.29515066 51.72132591

1 -19.27814331 14.30400687 48.68615142

1 -23.27685995 14.44535914 38.40758162

1 -19.62160288 10.93699498 46.85766813

1 -21.39709553 6.48078791 46.36060623

1 -20.43223095 2.35143645 44.40342217

1 -15.81528176 6.14584511 38.85952420

1. Fei, R., *Novel Platinum (Pt)-Vandetanib Hybrid Compounds: Design, Synthesis and Investigation of Anti-cancer Activity and Mechanism of Action*. 2016: The Chinese University of Hong Kong (Hong Kong).
2. Knowles, P.P., et al., *Structure and chemical inhibition of the RET tyrosine kinase domain.* Journal of biological chemistry, 2006. **281**(44): p. 33577-33587.
3. Smith, O., et al., *Control of stereogenic oxygen in a helically chiral oxonium ion.* Nature, 2023. **615**(7952): p. 430-435.
4. Brocklesby, K.L., et al., *An alternative synthesis of Vandetanib (Caprelsa™) via a microwave accelerated Dimroth rearrangement.* Tetrahedron letters, 2017. **58**(15): p. 1467-1469.
5. Al-Ghusn, A.I., et al., *Vandetanib.* Profiles of Drug Substances, Excipients and Related Methodology, 2023. **48**: p. 109-134.
6. Tung, R., *Vandetanib derivatives*, C.-R.T.R. APPLICATIONS, Editor. 2013: US.
